# Indole‐3‐Carboxaldehyde Inhibits Inflammatory Response and Lipid Accumulation in Macrophages Through the miR‐1271‐5p/HDAC9 Pathway

**DOI:** 10.1111/jcmm.70263

**Published:** 2024-12-19

**Authors:** Wei Luo, Jun Meng, Xiao‐Hua Yu, Zi‐Zhen Zhang, Gang Wang, Jin He

**Affiliations:** ^1^ The First Affiliated Hospital, Department of Cardiology, Hengyang Medical School University of South China Hengyang Hunan China; ^2^ The First Affiliated Hospital, Department of Function, Hengyang Medical School University of South China Hengyang Hunan China; ^3^ Institute of Clinical Medicine The Second Affiliated Hospital of Hainan Medical University Haikou Hainan China; ^4^ School of Medical and Pharmacological Technology Hunan Polytechnic of Environment and Biology Hengyang Hunan China

**Keywords:** atherosclerosis, HDAC9, ICA, inflammatory response, lipid accumulation, miR‐1271‐5p

## Abstract

Indole‐3‐carboxaldehyde (ICA), a microbiota‐derived tryptophan metabolite, has been reported to protect against atherosclerosis. However, the molecular mechanisms for its atheroprotective effect remain largely unknown. This study aimed to explore the influence of ICA on lipid accumulation and inflammatory response in THP‐1 macrophage‐derived foam cells. Our results showed that administration of ICA upregulated the expression of miR‐1271‐5p, ATP binding cassette transporter A1 (ABCA1) and ABCG1, downregulated histone deacetylase 9 (HDAC9) expression and inhibited macrophage lipid accumulation. ICA treatment also facilitated macrophage polarisation to the M2 phenotype and alleviated inflammatory response, as evidenced by decreased IL‐6 levels and increased IL‐10 levels. HDAC9 was identified as a direct target of miR‐1271‐5p. HDAC9 overexpression or miR‐1271‐5p knockdown decreased the effect of ICA on ABCA1 and ABCG1 expression as well as inflammatory response. Taken together, these results suggest that ICA can suppress lipid accumulation and mitigate inflammatory response in macrophages by activating the miR‐1271‐5p/HDAC9 signalling cascade, thereby providing new explanations for how ICA reduces atherosclerosis.

## 1 Introduction

Atherosclerosis is regarded as the major pathological basis of cardiovascular and cerebrovascular diseases, the first cause of death in the word [1]. Although the pathogenesis of atherosclerosis is complex and multifactorial, both macrophage lipid accumulation and inflammatory response play key roles in atherogenesis [2, 3]. After uptake of abundant modified lipoproteins such as oxidised low‐density lipoprotein (ox‐LDL), macrophages are transformed to foam cells, a hallmark of atherosclerosis [4]. ATP binding cassette transporter A1 (ABCA1) and G1 (ABCG1) mediates the efflux of intracellular cholesterol to apolipoprotein A‐I (apoA‐I) and high‐density lipoprotein (HDL), respectively. Studies from our group and others have demonstrated that promoting the expression of these two transporters can suppress lipid accumulation in macrophages and mitigate atherosclerosis in animal models [5, 6, 7]. Notably, macrophages can also secrete a variety of proinflammatory mediators, which contribute to lesion expansion [8]. Therefore, exploring a novel regulatory mechanism for macrophage lipid accumulation and inflammatory response is important to develop new therapeutic targets of atherosclerosis‐related diseases.

**FIGURE 1:**
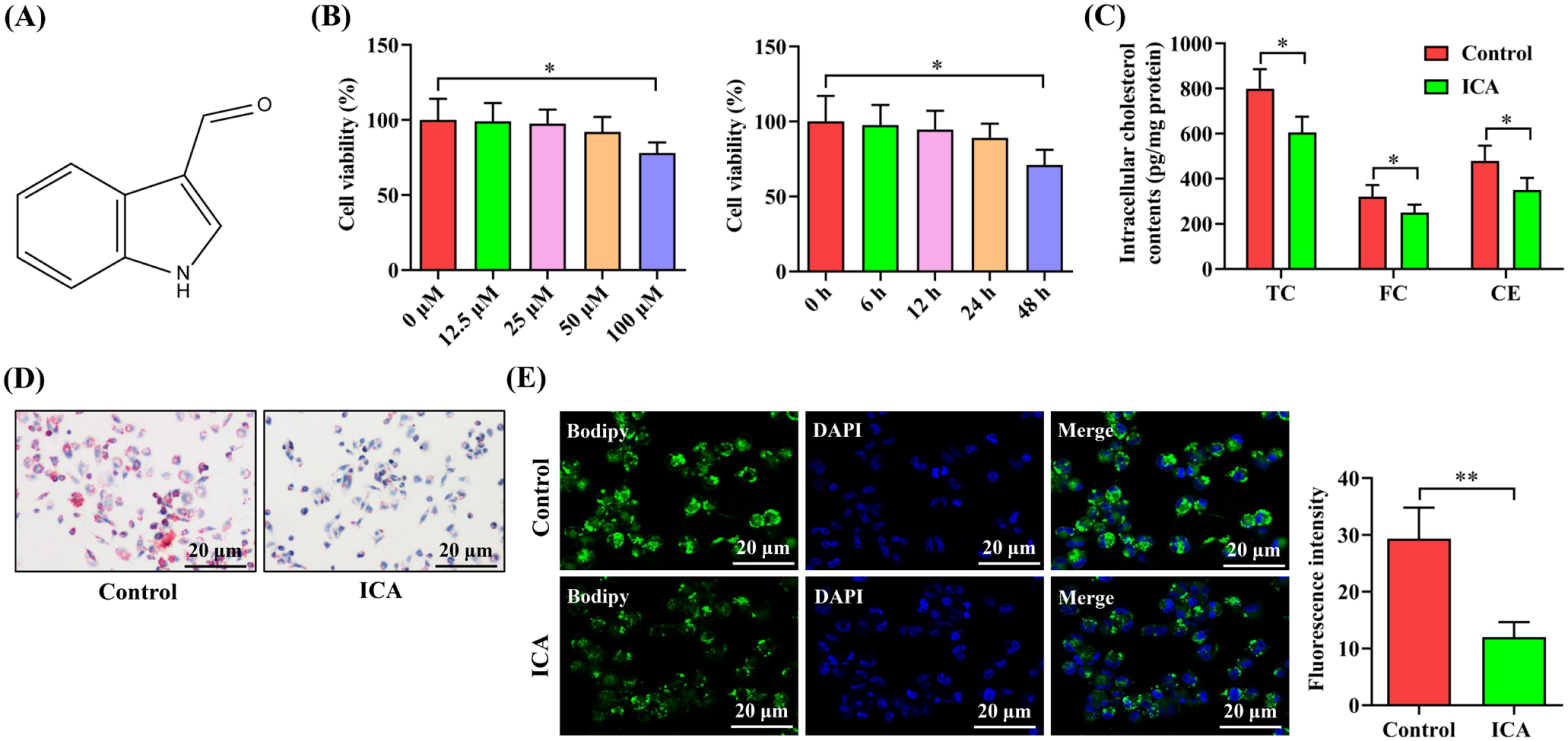
Inhibitory effect of ICA on macrophage lipid accumulation. (A) Chemical structure of ICA. (B) THP‐1 macrophage‐derived foam cells were treated with the indicated concentrations of ICA for 24 h or incubated with 50 μM ICA for different time points. CCK‐8 was applied to measure cell viability. (C–E) THP‐1 macrophage‐derived foam cells were exposed to ICA (50 μM) or DMSO for 24 h. (C) Detection of intracellular TC, FC and CE contents using HPLC. (D) Representative images of oil red O staining. (E) Representative images of Bodipy 493/503 staining and quantitative analysis. Data are expressed as mean ± SD from three independent experiments. *p < 0.05, **p < 0.01.

Gut microbiota‐derived metabolites not only contribute to host health but also participate in the occurrence and development of multiple diseases. In recent years, the relationship of microbial metabolites originating from amino acid with atherosclerosis has attracted a lot of attention [9, 10, 11]. Indole‐3‐carboxaldehyde (ICA, Figure 1A) is a microbiota‐derived metabolite from the dietary tryptophan, an essential amino acid for human body. Several lines of evidence have demonstrated that ICA can alleviate inflammation in chondrocytes and RAW264.7 cells [12, 13]. Plasma ICA concentration is lower in patients with advanced atherosclerosis [14]. Importantly, a recent study showed that administration of ICA decreases atherosclerotic burden in apolipoprotein E‐deficient mice by inhibiting vascular inflammation and oxidative stress [15]. However, the mechanisms for its atheroprotection is still largely unknown.

Histone deacetylases (HDACs) are known to catalyse the deacetylation of the acetylated lysine residues of histones, leading to compact chromatin structure and gene transcription repression. As one member of the subgroup IIa, HDAC9 expression is upregulated in human carotid plaques [16]. Deficiency of HDAC9 promotes cholesterol efflux from macrophages and reduces plaque area in atherosclerosis‐prone mice by enhancing the expression of ABCA1 and ABCG1 [17]. MicroRNAs (miRNAs) are short noncoding single‐stranded RNA molecules that can bind to 3′‐untranslated region (UTR) of target mRNAs for inhibiting gene expression. There is increasing evidence that dysregulation of miRNAs is closely related to lipid metabolism, inflammation and atherosclerosis [18, 19]. MiR‐1271‐5p is a recently discovered miRNA. It has been suggested that miR‐1271‐5p can alleviate cardiac injury in a mouse model of acute myocardial infarction [20]. However, it is unclear whether there is a direct interaction between miR‐1271‐5p and HDAC9.

Here, we found that gut microbiota‐derived tryptophan metabolite ICA‐suppressed lipid accumulation and inflammatory response by activating the miR‐1271‐5p/HDAC9 signalling pathway in THP‐1 macrophage‐derived foam cells. This finding provides novel mechanistical insights into the atheroprotective effect of ICA.

## 2 Materials and Methods

### 2.1 Cells, Reagents and Antibodies

Human monocyte line THP‐1 and 293 T cells were provided by American Type Culture Collection (Rockville, MD, USA). ICA was purchased from MCE (Shanghai, China) and dissolved in dimethyl sulfoxide (DMSO). Fetal bovine serum (FBS), phorbol‐12‐myristate acetate (PMA), apoA‐I, actinomycin D (ActD), DMSO and DAPI were obtained from Sigma‐Aldrich (St. Louis, MO, USA). Rabbit antibodies against CD36, scavenger receptor class A (SR‐A), sterol regulatory element‐binding protein 2 (SREBP2), ABCG1 and HDAC9 were supplied by Abcam (Cambridge, UK). Rabbit antibodies against CD86, inducible nitric oxide synthase (iNOS), CD206, arginase‐1 (Arg‐1) and β‐actin were provided by Proteintech (Chicago, IL, USA). The CCK‐8 cell proliferation and cytotoxicity assay kit (Solarbio, Beijing, China), mouse antibody against 3‐hydroxy‐3‐methylglutaryl‐CoA reductase (HMGCR, Abcam), rabbit antibody against ABCA1 (ThermoFisher, Waltham, MA, USA) and horseradish peroxidase (HRP)‐conjugated goat antirabbit or mouse IgG (Beyotime, Shanghai, China) were obtained as indicated.

### 2.2 Cell Culture and ICA Treatment

THP‐1 monocytes were maintained in RPMI 1640 medium (Solarbio) supplemented with 10% FBS and 2% penicillin–streptomycin in a humidified incubator containing 5% CO_2_ at 37°C. The cells were differentiated into macrophages by using 160 nM PMA for 24 h. Then, THP‐1 macrophages were converted to lipid‐rich foam cells after 48 h of incubation with 50 μg/mL of ox‐LDL (Yiyuan Biotechnology, Guangzhou, China). To investigate the effect of ICA on cell viability, THP‐1 macrophage‐derived foam cells were treated with 0, 12.5, 25, 50 or 100 μM ICA for 24 h or incubated with 50 μM ICA for 0, 6, 12, 24 or 48 h. In other experiments, THP‐1 macrophage‐derived foam cells were exposed to ICA (50 μM) or the vehicle (DMSO) for 24 h.

### 2.3 Cell Viability Assessment

THP‐1 macrophage‐derived foam cells were seeded into 96‐well plates (2 × 10^6^ cells per well). After treatment as described above, CCK‐8 reagent (10 μL) was added to each well. Cells were then cultured at 37°C for 2 h. The absorbance of each well was detected at 450 nm for the calculation of cell viability.

### 2.4 High‐Performance Liquid Chromatography (HPLC)

The levels of intracellular total cholesterol (TC), free cholesterol (FC) and cholesterol ester (CE) were measured by HPLC. After washing three times with PBS, THP‐1 macrophage‐derived foam cells were broken by an ultrasonic processor on the ice. Then, protein concentrations in cell lysates supernatants were measured by the BCA Protein Assay Kit (Beyotime, Shanghai, China). Cellular lipids were extracted by n‐hexane‐isopropanol (3:2, V/V) and then dissolved in isopropanol (50 mg/mL). The TC content was measured by adding 0.4 U cholesterol oxidase combined with 0.4 U cholesterol esterase, and FC content was examined by using 0.4 U cholesterol oxidase. The CE content was calculated by subtracting FC from TC.

### 2.5 Oil Red O Staining

THP‐1 macrophage‐derived foam cells were washed with PBS after treating with DMSO or ICA. Subsequently, 4% paraformaldehyde was added into each well for fixation. Ten min later, cells were stained with 0.5% oil red O solution (Solarbio), and then washed with 85% isopropanol. Haematoxylin was used for counterstaining, and cells were photographed under a microscope.

### 2.6 Bodipy 493/503 Staining

After fixation with 4% paraformaldehyde for 15 min, THP‐1 macrophage‐derived foam cells were stained with 1 mg/mL Bodipy 493/503 (ThermoFisher). After 30 min of staining, DAPI was added into each well and then incubated at 37°C to identify nuclei. After 30 min, staining was captured with a Leica STELLARIS five laser scanning confocal microscope.

### 2.7 NBD‐Cholesterol Efflux Assay

After the indicated treatment, THP‐1 macrophage‐derived foam cells were planked in six‐well plates and incubated with 1 μg/mL NBD‐cholesterol (Invitrogen, Carlsbad, CA, USA) at 37°C for 4 h. Cells were washed with PBS. Then, apoA‐I (25) or 50 μg/mL of HDL (Yiyuan Biotechnology) was added and incubated for another 4 h at 37°C. The medium was collected, and cells were lysed. The fluorescence intensity of the medium and cell lysates was detected using a microplate spectrophotometer. NBD‐cholesterol efflux capacity was expressed as the percentage of fluorescence intensity in the medium to total fluorescence intensity (medium + cell lysates).

### 2.8 Dil‐Ox‐LDL Uptake Assay

THP‐1 macrophage‐derived foam cells treated with DMSO or ICA were washed twice with PBS. Dil‐ox‐LDL (10 μg/mL) was added and incubated at 37°C for 4 h, followed by treatment with DAPI at 37°C for 30 min. After washing with PBS, cells were photographed using a fluorescence microscope (TS2R‐FL, Nikon, Japan).

### 2.9 MiR‐1271‐5p Mimic/Inhibitor Transfection and HDAC9 Overexpression Plasmid Construction

For overexpression or knockdown of miR‐1271‐5p, THP‐1 macrophage‐derived foam cells were transfected with 50 nM of miR‐1271‐5p mimic, inhibitor or their respective negative controls (Ribobio, Guangzhou, China) using Lipofectamine 3000 reagent (Invitrogen) according to the manufacturer's protocols. The cDNA sequence of HDAC9 was synthesised from mRNA of THP‐1 macrophages. The product was inserted into the pcDNA3.1 vector (GenePharma, Shanghai, China) to construct plasmids with HDAC9 overexpression (pcDNA‐HDAC9). Afterwards, 60 nM of pcDNA3.1‐HDAC9 or empty vector as a negative control (pcDNA‐NC) were transfected into THP‐1 macrophage‐derived foam cells at the aid of Lipofectamine 3000 reagent. Transfection efficiency was determined after 48 h of transfection by detecting the expression of miR‐1271‐5p and HDAC9.

### 2.10 Bioinformatics Analysis and Luciferase Reporter Assay

The interaction between miR‐1271‐5p and HDAC9 3′‐UTR was predicted by the TargetScan (http://www.targetscan.org/), miRDB (http://mirdb.org/miRDB/), miRanda (http://www.microrna.org/microrna/home.do) and PicTar (http://www.pictar.org/) databases. The RNAhybrid database (http://bibiserv.techfak.Uni‐bielefeld.de/rnahybrid/submission.html) was employed to evaluate the free energy score. The entire 3′‐UTR of HDAC9 was subcloned into the pGL3 vector (Promega, Madison, WI, USA), creating the HDAC9‐WT construct. The miR‐1271‐5p binding site within the 3′‐UTR of HDAC9 was mutated to establish the corresponding mutant plasmids (HDAC9‐Mut). The 293 T cells were plated into 24‐well plates (2 × 10^6^ cells/well) and cultured in DMEM medium (Gibco, Grand Island, NE, USA) containing 10% FBS. These constructs (50 ng) were cotransfected into 293 T cells with 50 nM of miR‐1271‐5p mimic or mimic control using Lipofectamine 3000 reagent. After 48 h, cells were harvested to measure firefly and Renilla luciferases using the Dual‐Luciferase Reporter Assay System (Promega). To evaluate the impact of ICA, 293 T cells were treated with the pGL3 vector containing HDAC9 3′‐UTR (pGL3‐HDAC9 3′‐UTR) or empty vector (pGL3‐NC) in combination with ICA or DMSO. The luciferase activity was then examined as described above.

The effect of ICA on the promoter activity of HDAC9 was measured by luciferase reporter assay as previously described [21]. Briefly, the luciferase reporter plasmids containing human HDAC9 promoter region of 2000 bp (pGL3‐HDAC9) were constructed by Genechem (Shanghai, China), with the empty pGL3 vector as a negative control (pGL3‐NC). These plasmids (0.5 μg) were transfected into 293 T cells by using Lipofectamine 3000 reagent. Subsequently, cells were treated with ICA or DMSO to measure the luciferase activity.

### 2.11 HDAC9 mRNA Stability Assay

THP‐1 macrophage‐derived foam cells were treated with ICA or DMSO for 24 h and then incubated with ActD (5 μg/mL) for another 24 h to block gene transcription. After 0, 2, 4 and 8 h of ActD incubation, cells were harvested to detect HDAC9 mRNA expression levels using quantitative real‐time polymerase chain reaction (qRT‐PCR).

### 2.12 Enzyme‐Linked Immunosorbent Assay (ELISA)

After the indicated treatment, the cell culture supernatant was collected. The levels of interleukin (IL)‐6 and (IL)‐10 were measured using the commercial ELISA kits (Solarbio) following the manufacturer's protocol.

### 2.13 qRT‐PCR

Total RNA was isolated using the TRIzol reagent (Invitrogen) from THP‐1 macrophage‐derived foam cells, and cDNA was synthesised using the Superscript First‐Strand cDNA Synthesis Kit (Invitrogen). The qRT‐PCR was then performed by using SYBR Green Real‐Time PCR Master Mix (Promega) on an ABI 7900HT Fast Real‐Time PCR System (Applied Biosystems, Foster City, CA, USA). U6 was used as an internal control for the normalisation of miRNAs and GAPDH for protein‐coding genes. The relative expression levels of target genes were calculated using the 2^−ΔΔ*Ct*^ method. The primers used for qRT‐PCR are designed and synthesised by Sangon Biotech (Shanghai, China), and their sequences are described in Table S1.

### 2.14 Western Blot

The RIPA lysis buffer containing 0.1 mmol/L phenylmethanesulfonyl fluoride (Beyotime) was used to lyse THP‐1 macrophage‐derived foam cells. Protein concentration was determined by a BCA Protein Assay Kit (Beyotime). Protein samples were separated by sodium dodecyl sulfate‐polyacrylamide gel electrophoresis and then transferred to polyvinylidene difluoride membranes (Millipore, Billerica, MA, USA). After blockade with 5% skim milk for 2 h at room temperature, the membranes were incubated with the primary antibodies against CD36 (ab133625, 1:1000), SR‐A (ab271070, 1:1000), HMGCR (ab242315, 1:1000), SREBP2 (ab30682, 1:800), ABCA1 (PA1‐16789, 1:500), ABCG1 (ab52617, 1:800), HDAC9 (ab109446, 1:1000), CD86 (13395‐1‐AP, 1:1000), iNOS (18985‐1‐AP, 1:1000), CD206 (18704‐1‐AP, 1:1000), Arg‐1 (16001‐1‐AP, 1:1000) or β‐actin (20536‐1‐AP, 1:3000) at 4°C overnight. The membranes were washed three times with TBST for 10 min each time and then incubated with HRP‐conjugated secondary antibodies (1:3000) for 2 h at room temperature with sustained shaking. Protein bands were visualised using the BeyoECL Plus kit (Beyotime) on Tanon 5500 (Shanghai, China), with β‐actin as an internal control.

### 2.15 Statistical Analysis

Statistical analysis was performed with GraphPad Prism 8.0 software (San Diego, CA, USA). All results were expressed as the mean ± standard deviation (SD) from three independent experiments. An unpaired two‐tailed Student *t*‐test was used to comparing the data between two groups. The one‐way ANOVA followed by Tukey's multiple comparison test was applied to compare the data among multiple groups. A value of *p* < 0.05 was considered to indicate a statistically significant difference.

## 3 Results

### 3.1 ICA Attenuates Intracellular Lipid Accumulation in THP‐1 Macrophage‐Derived Foam Cells

CCK‐8 was employed to explore the impact of ICA on cell viability to determine the optimal treatment concentration and time for ICA. To this end, THP‐1 macrophage‐derived foam cells were treated with various concentrations of ICA (0, 12.5, 25, 50 or 100 μM) for 24 h. The CCK‐8 results demonstrated that treatment with 100 μM ICA significantly reduced cell viability, while ICA had no influence on the viability at concentrations below 50 μM (Figure 1B). Subsequently, cells were exposed to 50 μM ICA for different durations (0, 6, 12, 24 or 48 h). There was no cytotoxicity when cells were incubated with 50 μM ICA for 6–24 h (Figure 1B). Therefore, 50 μM of ICA was used to treat THP‐1 macrophage‐derived foam cells for 24 h in the subsequent experiments. Macrophage lipid accumulation is regarded as a critical determinant for foam cell formation and atherogenesis [22]. To evaluate the effect of ICA on macrophage lipid accumulation, THP‐1 macrophage‐derived foam cells were incubated with ICA (50 μM) or DMSO for 24 h. The levels of intracellular TC, FC and CE were significantly decreased after ICA incubation, as evidenced by the HPLC assay (Figure 1C). The oil red O staining results demonstrated that the intracellular lipid droplets in ICA group were smaller and fewer than those in control group (Figure 1D). The Bodipy 493/503 staining results further verified that ICA could markedly suppress intracellular lipid deposition (Figure 1E). These findings reveal an inhibitory effect of ICA on lipid accumulation in macrophages.

### 3.2 ICA Promotes ABCA1 and ABCG1 Expression and Cholesterol Efflux in THP‐1 Macrophage‐Derived Foam Cells

**FIGURE 2:**
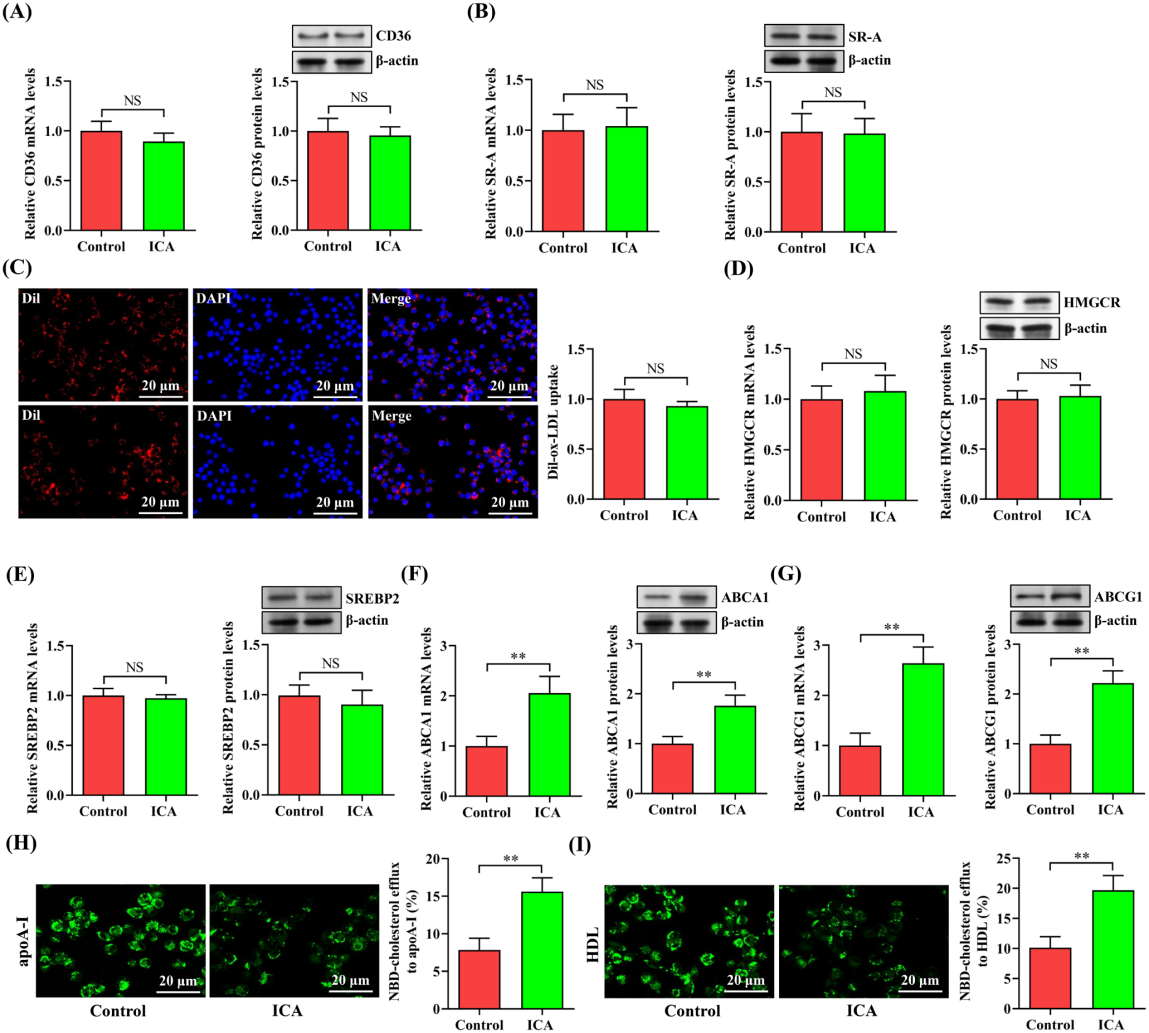
ICA promotes macrophage cholesterol efflux by enhancing ABCA1 and ABCG1 expression. (A–I) THP‐1 macrophage‐derived foam cells were treated with ICA (50 μM) or DMSO for 24 h. (A, B) The expression of CD36 and SR‐A was determined by qRT‐PCR and western blot. (C) Representative photomicrographs and quantification of Dil‐ox‐LDL uptake. (D, E) Detection of HMGCR and SREBP2 expression using qRT‐PCR and western blot. (F, G) Qrt‐PCR and western blot analyses of ABCA1 and ABCG1 expression. (H, I) Representative photomicrographs of NBD‐cholesterol burden and quantitative analyses of cholesterol efflux to apoA‐I and HDL. Data shown are mean ± SD from three independent experiments. **p < 0.01. NS indicates not significant.

Macrophage lipid accumulation is attributed by disorders in intracellular cholesterol homeostasis, which is maintained by cholesterol influx, biosynthesis and efflux [4]. To figure out the potential molecular mechanisms for ICA‐mediated prevention of macrophage lipid accumulation, we tested the impact of ICA on these biological processes. We found that ICA administration did not alter the expression of CD36 (Figure 2A) and SR‐A (Figure 2B), two critical factors that are involved in the internalisation of modified lipoproteins. Consistently, there was no significant difference in Dil‐ox‐LDL uptake between ICA group and control group (Figure 2C). Also, ICA had no effect on the expression of HMGCR (Figure 2D) and SREBP2 (Figure 2E), both of which play a key role in stimulating endogenous cholesterol synthesis. ABCA1 is known to facilitate the export of intracellular cholesterol to lipid‐poor apoA‐I, while ABCG1 mediates cholesterol transport to HDL [23]. Interestingly, treatment with ICA led to a significant increase in the mRNA and protein levels of ABCA1 (Figure 2F) and ABCG1 (Figure 2G). Accordingly, ICA enhanced the capability of NBD‐cholesterol efflux to apoA‐I (Figure 2H) and HDL (Figure 2I). Together, these observations indicate that ICA protects against macrophage lipid accumulation by promoting ABCA1 and ABCG1‐dependent cholesterol efflux.

### 3.3 HDAC9 Is Involved in Induction of ABCA1 and ABCG1 Expression by ICA

**FIGURE 3:**
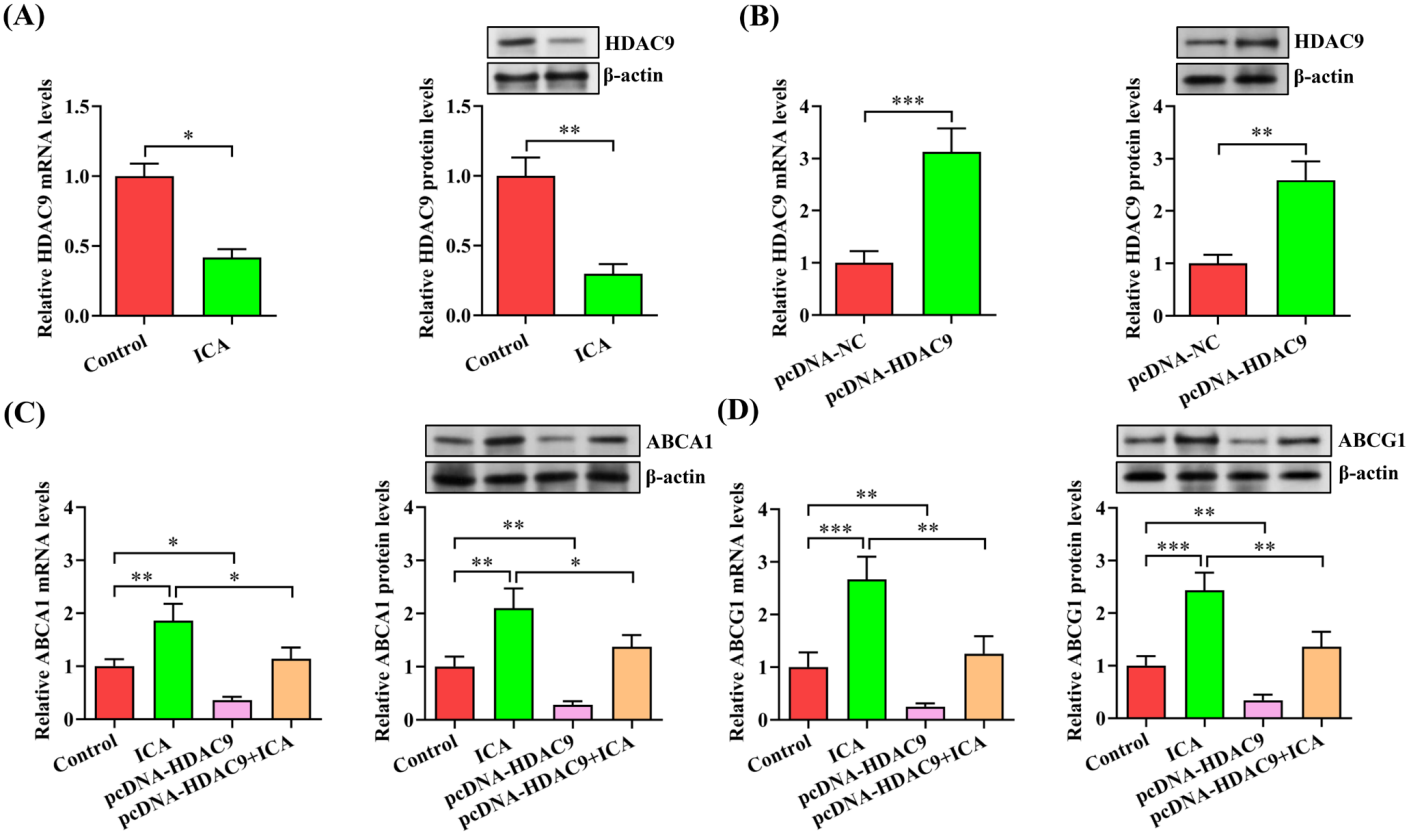
Involvement of HDAC9 in ICA‐induced upregulation of ABCA1 and ABCG1 expression. (A) After 24 h of incubation with ICA (50 μM) or DMSO, qRT‐PCR and western blot were used to examine HDAC9 expression in THP‐1 macrophage‐derived foam cells. (B) Measurement of HDAC9 expression using qRT‐PCR and western blot in THP‐1 macrophage‐derived foam cells transfected with 60 nM of pcDNA‐HDAC9 or pcDNA‐NC for 48 h. (C, D) THP‐1 macrophage‐derived foam cells were transfected with pcDNA‐HDAC9 (60 nM) for 48 h and then treated with or without ICA (50 μM) for an additional 24 h. Data are presented as mean ± SD from three independent experiments. *p < 0.05, **p < 0.01, ***p < 0.001.

HDAC9 is known to function as a repressor of ABCA1 and ABCG1 gene transcription [17]. To determine whether ICA‐induced upregulation of ABCA1 and ABCG1 expression is mediated by HDAC9, we first examined the expression of this deacetylase in THP‐1 macrophage‐derived foam cells treated with ICA or DMSO. As expected, decreased levels of HDAC9 mRNA and protein were observed in ICA group compared with control group (Figure 3A). We then overexpressed HDAC9 by using the pcDNA3.1 vector (Figure 3B), which was transfected into THP‐1 macrophage‐derived foam cells prior to ICA treatment. ICA‐enhanced expression of ABCA1 and ABCG1 was reversed by HDAC9 overexpression (Figure 3C,D), suggesting that ICA upregulates ABCA1 and ABCG1 expression in a HDAC9‐dependent manner.

### 3.4 ICA Inhibits HDAC9 Expression by Increasing miR‐1271‐5p Levels

**FIGURE 4:**
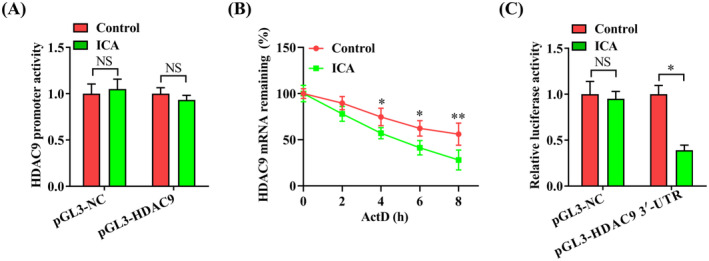
ICA reduces the stability of HDAC9 mRNA. (A) 293 T cells transfected with pGL3‐HDAC9 or pGL3‐NC were treated with ICA (50 μM) or DMSO for 24 h. Cell lysates were used to detect the luciferase activity. (B) The half‐life of HDAC9 mRNA in THP‐1 macrophage‐derived foam cells pretreated with ICA (50 μM) or DMSO for 24 h were determined with the addition of ActD. (C) 293 T cells transfected with pGL3‐HDAC9 3′‐UTR or pGL3‐NC were incubated with ICA (50 μM) or DMSO. The luciferase activity was then examined after 24 h. Data represent the mean ± SD of three independent experiments. *p < 0.05, **p < 0.01. NS indicates not significant.

To explore how ICA represses HDAC9 expression, human HDAC9 promoter luciferase fusion gene constructs were applied to evaluate the influence of ICA on the HDAC9 promoter activity. Our results showed that there was no significant alteration in the HDAC9 promoter activity between ICA group and control group (Figure 4A), thereby excluding the effect of ICA on HDAC9 gene transcription. We then used ActD to block gene transcription for evaluating the mRNA stability of HDAC9. We found that administration of ICA reduced the half‐life of HDAC9 mRNA in THP‐1 macrophage‐derived foam cells (Figure 4B), revealing that ICA can diminish the stability of HDAC9 mRNA. Finally, we constructed the luciferase reporter plasmid containing human HDAC9 3′‐UTR region, which was transfected into 293 T cells in the presence or absence of ICA. As illustrated in Figure 4C, the luciferase activity was significantly decreased in ICA group when compared to control group. Collectively, these findings suggest that ICA modulates HDAC9 expression at the posttranscriptional level partly by attenuating HDAC9 mRNA stability via its 3′‐UTR sequence.

**FIGURE 5:**
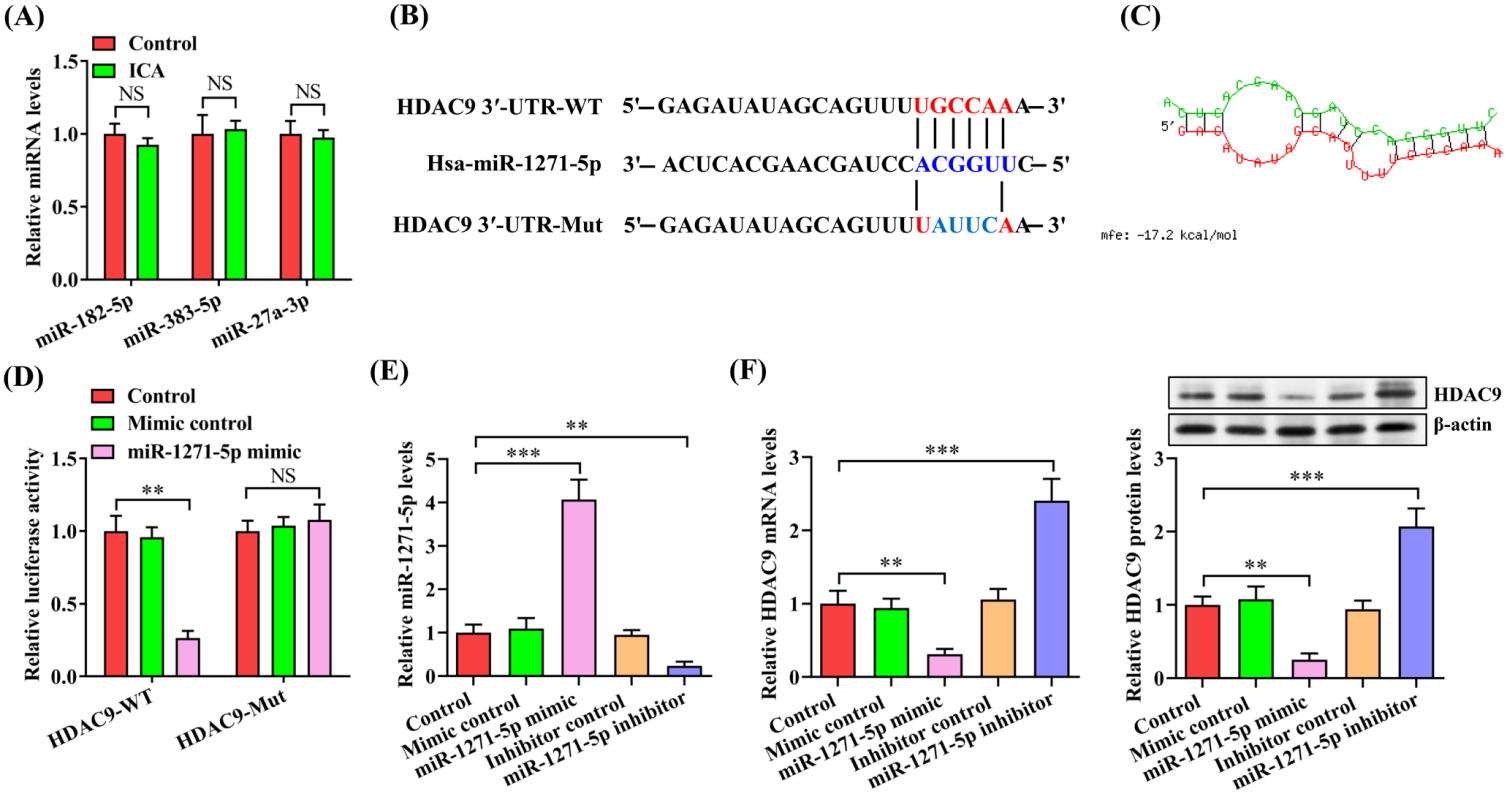
Identification of HDAC9 as a direct target of miR‐1271‐5p. (A) After treatment of THP‐1 macrophage‐derived foam cells with ICA (50 μM) or DMSO for 24 h, qRT‐PCR was employed to analyse the expression of miR‐182‐5p, miR‐383‐5p and miR‐27a‐3p. (B) The binding site between miR‐1271‐5p and HDAC9 3′‐UTR and corresponding mutation. (C) Free energy score for the interaction of miR‐1271‐5p with HDAC9 3′‐UTR. (D) The luciferase activity was detected in 293 T cells transfected with HDAC9‐WT or HDAC9‐Mut plasmids plus 50 nM of miR‐1271‐5p mimic or mimic control for 48 h. (E, F) THP‐1 macrophage‐derived foam cells were transfected with miR‐1271‐5p mimic/inhibitor or their negative controls (50 nM) for 48 h. (E) QRT‐PCR analysis of miR‐1271‐5p expression. (F) Detection of HDAC9 expression using qRT‐PCR and western blot. Data are presented as mean ± SD from three independent experiments. **p < 0.01, ***p < 0.001. NS indicates not significant.

MiRNAs are known to serve as critical regulators of mRNA stability by binding to 3′‐UTR of their target genes [24]. Several lines of evidence have demonstrated that HDAC9 can be directly regulated by miRNAs, such as miR‐182‐5p, miR‐383‐5p and miR‐27a‐3p [25, 26, 27]. However, treatment of THP‐1 macrophage‐derived foam cells with ICA did not alter the expression of these miRNAs (Figure 5A). We thus inferred that specific miRNAs may mediate the dysregulated HDAC9 expression. We predicted the putative miRNAs containing the binding sites of HDAC9 by using four online databases: TargetScan, miRDB, miRanda and PicTar. Overlap analysis showed that miR‐1271‐5p contained a highly conserved consequence targeting HDAC9 3′‐UTR (Figure 5B). Meanwhile, the RNAhybrid database revealed a lower free energy score for the interaction of miR‐1271‐5p with HDAC9 3′‐UTR (−17.2 kcal/mol), suggesting that miR‐1271‐5p can combine stably with HDAC9 3′‐UTR (Figure 5C). To confirm the bioinformatics prediction results, we created luciferase reporter plasmids containing human HDAC9 3′‐UTR fragment with the miR‐1271‐5p target site (HDAC9‐WT) or the mutant miR‐1271‐5p target site (HDAC9‐Mut). These constructs were transfected into 293 T cells alongside miR‐1271‐5p mimic or mimic control for detection of the luciferase activity. As anticipated, miR‐1271‐5p mimic reduced the luciferase activity of HDAC9‐WT but not HDAC9‐Mut (Figure 5D). Then, THP‐1 macrophage‐derived foam cells were transfected with miR‐1271‐5p mimic, inhibitor or their negative controls, showing a high transfection efficiency (Figure 5E). Importantly, miR‐1271‐5p overexpression decreased, while miR‐1271‐5p knockdown elevated, the mRNA and protein levels of HDAC9 (Figure 5F). The above results validate HDAC9 as a direct target of miR‐1271‐5p.

**FIGURE 6:**
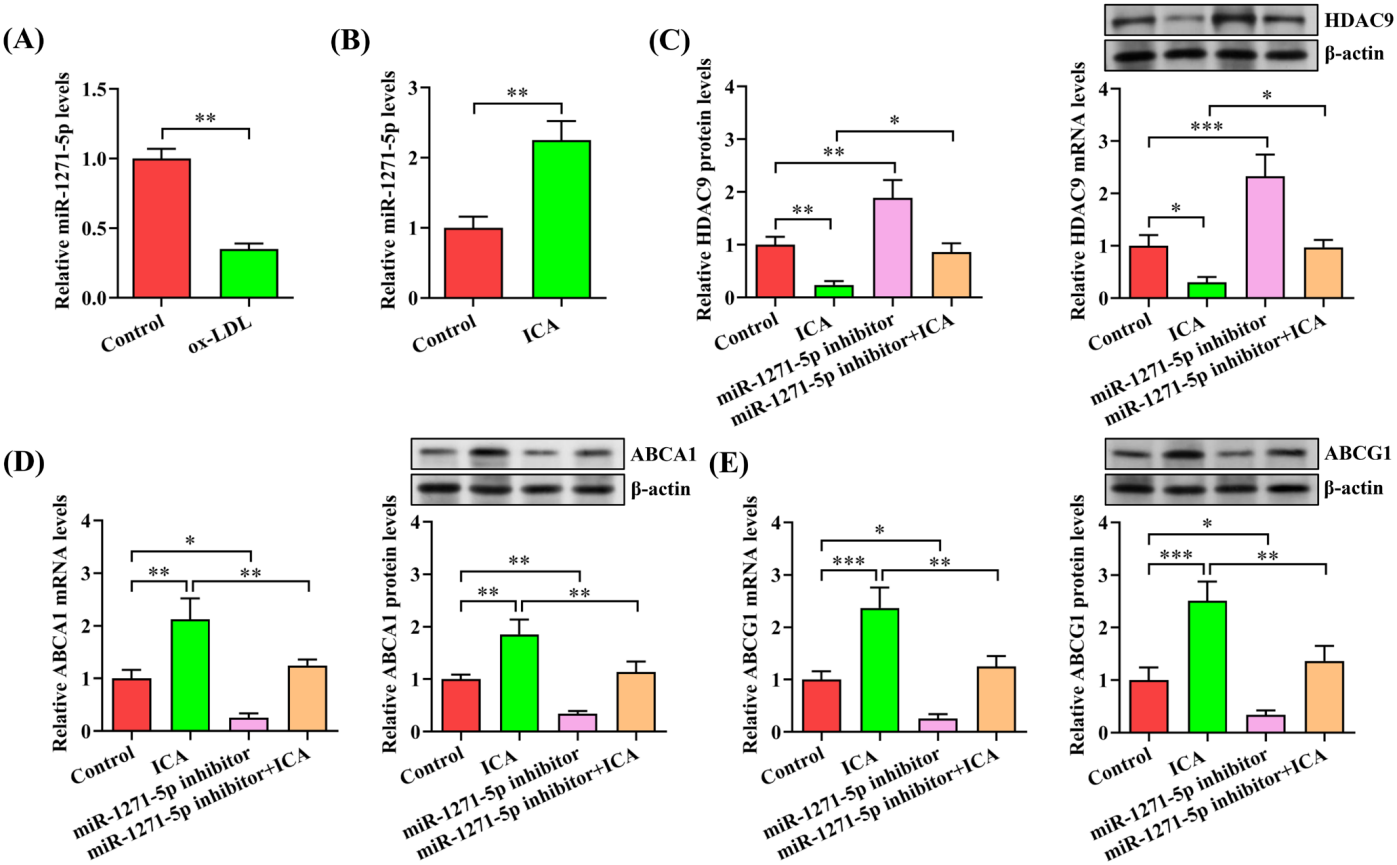
MiR‐1271‐5p is required for ICA‐regulated expression of HDAC9, ABCA1 and ABCG1. (A) QRT‐PCR assay of miR‐1271‐5p expression in THP‐1 macrophages loaded with or without ox‐LDL. (B) THP‐1 macrophage‐derived foam cells were treated with ICA (50 μM) or DMSO for 24 h, followed by qRT‐PCR analysis of miR‐1271‐5p expression. (C–E) THP‐1 macrophage‐derived foam cells were transfected with miR‐1271‐5p inhibitor (50 nM) for 48 h and then treated with 50 μM ICA for an additional 24 h. The expression of HDAC9, ABCA1 and ABCG1 was determined by qRT‐PCR and western blot. The results are presented as mean ± SD from three independent experiments. *p < 0.05, **p < 0.01, ***p < 0.001.

To verify the role of miR‐1271‐5p in ICA‐regulated expression of HDAC9, ABCA1 and ABCG1, we first detected miR‐1271‐5p expression in THP‐1 macrophages loaded with ox‐LDL using qRT‐PCR. Incubation with ox‐LDL led to a significant decrease in miR‐1271‐5p levels (Figure 6A), implying a link of miR‐1271‐5p to atherosclerosis. We subsequently tested the influence of ICA on miR‐1271‐5p expression and found that administration of ICA markedly augmented miR‐1271‐5p levels in THP‐1 macrophage‐derived foam cells (Figure 6B). Finally, THP‐1 macrophage‐derived foam cells were transfected with miR‐1271‐5p inhibitor, following which cells were treated with or without ICA. As demonstrated in Figure 6C, the decrease in HDAC9 mRNA and protein expression by ICA was prevented by miR‐1271‐5p inhibitor (Figure 6C). Pretreatment with miR‐1271‐5p inhibitor could also reverse ICA‐induced enhancement of ABCA1 and ABCG1 expression (Figure 6D,E). Together, these data indicate that ICA represses HDAC9 expression by enhancing miR‐1271‐5p levels and thus results in increased ABCA1 and ABCG1 expression.

### 3.5 ICA Inhibits Inflammatory Response Through the miR‐1271‐5p/HDAC9 Signalling Cascade

**FIGURE 7:**
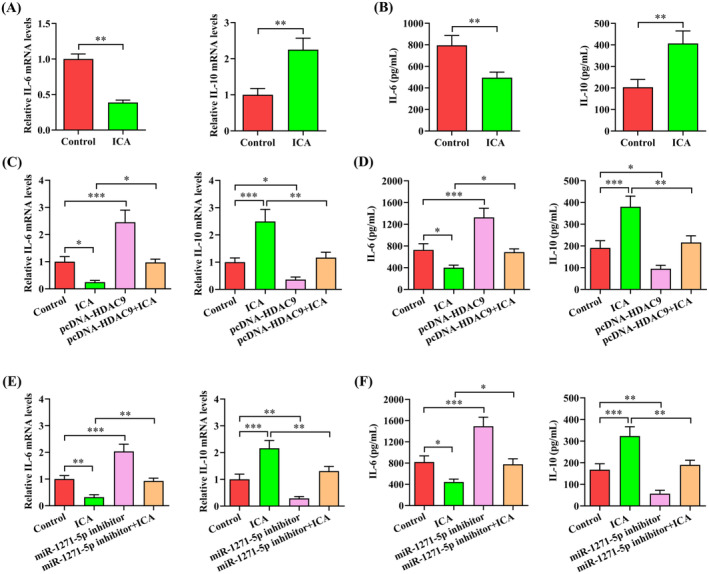
ICA represses inflammatory response via the miR‐1271‐5p/HDAC9 axis. (A and B) THP‐1 macrophage‐derived foam cells received the treatment with ICA (50 μM) or DMSO for 24 h. (A) The mRNA expression of IL‐6 and IL‐10 was determined by qRT‐PCR. (B) ELISA was applied to measure the levels of IL‐6 and IL‐10 in the cell culture supernatant. (C–F) THP‐1 macrophage‐derived foam cells were transfected with pcDNA‐HDAC9 (60 nM) or miR‐1271‐5p inhibitor (50 nM) for 48 h and then treated with ICA (50 μM) for 24 h. (C and E) QRT‐PCR analysis of IL‐6 and IL‐10 mRNA expression. (D, F) Detection of the levels of IL‐6 and IL‐10 in the cell culture supernatant using ELISA. Data are given as the mean ± SD from three independent experiments. *p < 0.05, **p < 0.01, ***p < 0.001.

It is well known that ox‐LDL can not only promote intracellular lipid accumulation but also trigger inflammatory response. ICA has been shown to suppress inflammatory response in multiple cell types [13, 28]. We next explored the impact of ICA on inflammatory response in THP‐1 macrophage‐derived foam cells and the underlying mechanism. Administration of ICA downregulated the mRNA expression of proinflammatory cytokine IL‐6, but upregulated the mRNA expression of anti‐inflammatory cytokine IL‐10 (Figure 7A). The ELISA analysis demonstrated that ICA also inhibited IL‐6 secretion and stimulated IL‐10 release (Figure 7B). Furthermore, pretreatment with pcDNA‐HDCA9 or miR‐1271‐5p inhibitor significantly reduced the effect of ICA on the expression and secretion of both cytokines (Figure 7C–F). These results imply that ICA activates the miR‐1271‐5p/HDAC9 pathway to mitigate inflammatory response.

### 3.6 ICA Induces M2 Macrophage Polarisation

**FIGURE 8:**
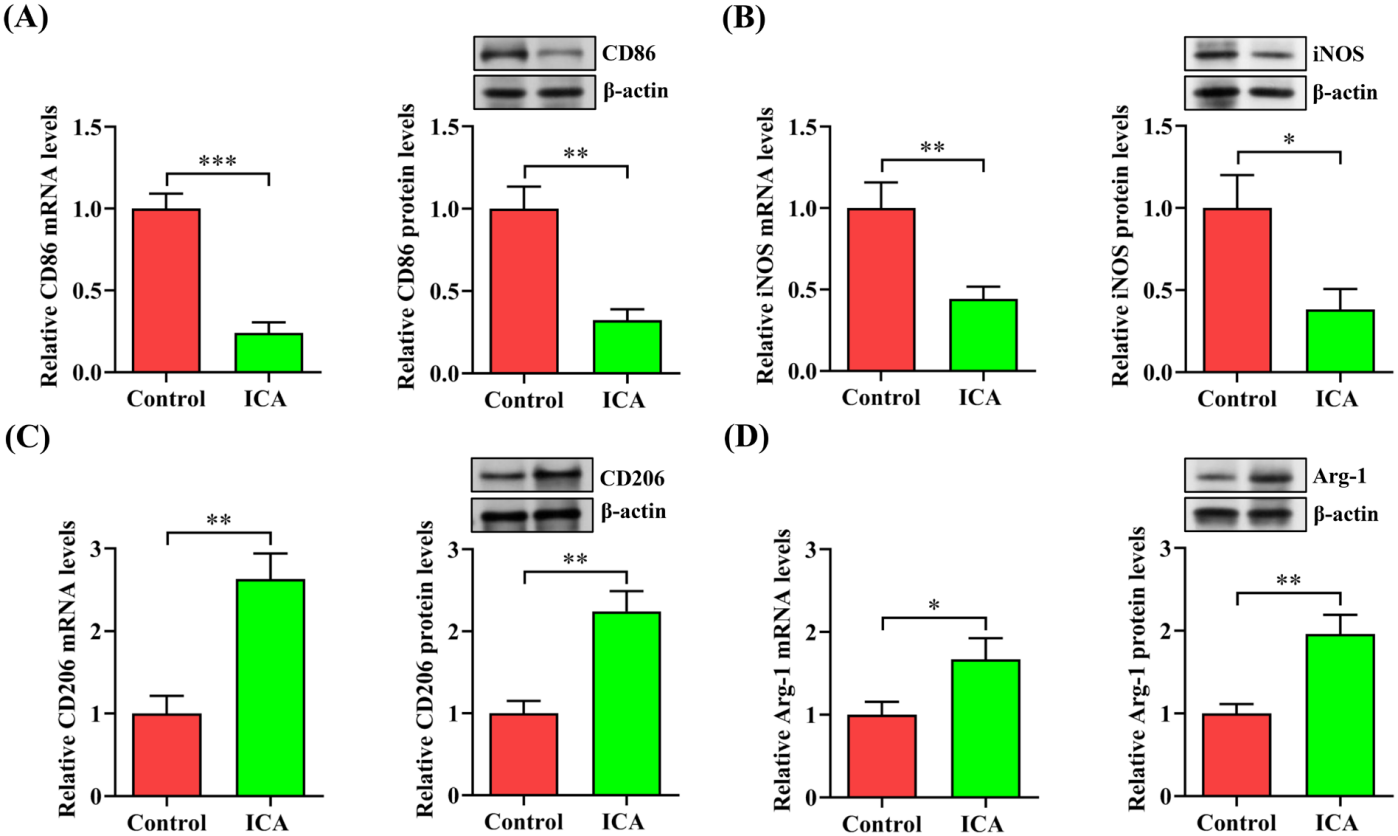
ICA facilitates the polarisation of macrophages to M2 phenotype. (A–D) THP‐1 macrophage‐derived foam cells were administrated with ICA (50 μM) or DMSO for 24 h. The expression of CD86, iNOS, CD206 and Arg‐1 was measured by qRT‐PCR and western blot. Data are expressed as the mean ± SD from three independent experiments. *p < 0.05, **p < 0.01, ***p < 0.001.

Macrophages can differentiate into two major subtypes depending on different stimuli. M1 macrophages secrete proinflammatory cytokines and exert a proatherogenic action, while M2 macrophages release anti‐inflammatory cytokine and inhibit the progression of atherosclerosis [29]. To determine the effect of ICA on macrophage polarisation, we measured the expression of M1 macrophage markers (CD86 and iNOS) as well as M2 macrophage markers (CD206 and Arg‐1) in THP‐1 macrophage‐derived foam cells. The qRT‐PCR and western blot results showed that the expression of CD86 and iNOS was significantly decreased in ICA‐treated cells (Figure 8A,B). In contrast, ICA treatment enhanced the levels of CD206 and Arg‐1 (Figure 8C,D). These data suggest that ICA can promote the polarisation of macrophages to M2 phenotype.

## 4 Discussion

Atherosclerosis, which is characterised by the accumulation of lipid and cholesterol deposits within the subendothelial space in the artery wall, is regarded as the major pathological basis for cardiovascular and cerebrovascular diseases. There is increasing evidence that gut microbiota can generate many dietary metabolites which are beneficial for host health, whereas the imbalance or dysbiosis of gut microbiota and derived metabolites is related to human diseases [30, 31, 32]. ICA is a gut microbiota‐derived tryptophan metabolite. In recent years, this metabolite has attracted researcher's attention as a new protective factor for intestinal disorders and metabolic diseases [13, 33]. Particularly, ICA has been reported to protect against atherosclerosis by repressing vascular inflammation and oxidative stress [15]. However, very little is known about its impact on macrophage lipid accumulation. By using THP‐1 macrophage‐derived foam cells, our study provides the first experimental evidence that administration of ICA greatly decreased intracellular cholesterol contents and reduced the number and size of lipid droplets. These findings indicate that prevention of macrophage lipid accumulation is another important mechanism by which ICA mitigates atherosclerosis.

Macrophage lipid accumulation leads to lipid‐enriched foam cell formation and atherosclerosis progression, which is attributed by disorders in intracellular cholesterol homeostasis. Three key steps, including cholesterol influx, biosynthesis and efflux, work in coordination to keep intracellular cholesterol homeostasis [4]. To uncover the molecular mechanism by which ICA inhibits macrophage lipid accumulation, we examined the effects of ICA on the expression of key factors involving these three processes in THP‐1 macrophage‐derived foam cells. Our results showed that ICA treatment dramatically upregulated ABCA1 and ABCG1 expression and had no impact on the levels of CD36, SR‐A and HMGCR and SREBP2. As the major contributors to cholesterol efflux, ABCA1 and ABCG1 are responsible for approximately 70% of cholesterol release from lipid‐rich macrophages, thereby playing a critical role in protecting against macrophage lipid accumulation and atherosclerosis [34, 35]. In further studies, we found that ICA promoted the efflux of NBD‐cholesterol from THP‐1 macrophage‐derived foam cells to apoA‐I and HDL, consistent with incremental ABCA1 and ABCG1 expression. Thus, ICA suppresses lipid accumulation in macrophages by enhancing ABCA1‐ and ABCG1‐dependent cholesterol export.

HDAC9, an important deacetylase, inhibits gene transcription by performing histone deacetylation. Increased HDAC9 expression is observed in human carotid, aortic and femoral plaques [16]. As a genetic variant in HDAC9 gene, rs2107595 is closely associated with the severity of coronary atherosclerosis in a Chinese Han population [36]. Inhibition of HDAC9 reduces lesion size throughout atherosclerotic aortas and increases plaque stability in animal models [37, 38, 39]. In addition, HDAC9 deletion enhances the expression of ABCA1 and ABCG1 by promoting H3, H4 and H3K9 acetylation at their promoters [17]. MiR‐1271‐5p is a miRNA with multiple biological effects. It has been reported that M2 macrophage‐derived exosomes carrying miR‐1271‐5p inhibits cardiomyocyte apoptosis and facilitates cardiac repair in a mouse model of acute myocardial infarction [20]. Circ_0007386 sponges miR‐1271‐5p to induce vascular smooth muscle cell apoptosis, leading to aggravation of thoracic aortic dissection [40]. These results imply miR‐1271‐5p as a cardiovascular protective factor. Here, we identified HDAC9 as a direct target of miR‐1271‐5p. ICA treatment downregulated HDAC9 expression but augmented miR‐1271‐5p levels in THP‐1 macrophage‐derived foam cells. Moreover, ICA‐enhanced ABCA1 and ABCG1 expression was reversed by HDAC9 overexpression or miR‐1271‐5p silencing, suggesting that ICA increases the expression of both transporters by activating the miR‐1271‐5p/HDAC9 signalling cascade.

The macrophages are a major cell type within the atherosclerotic plaques. M1 macrophages are predominantly distributed in the shoulders of vulnerable plaques and have weak abilities to transport and remove lipids. They can secrete proinflammatory cytokines and accelerate the development of atherosclerosis. Conversely, M2 macrophages are mainly located in the region far from the lipid core and accumulate less lipids. These cells release anti‐inflammatory cytokines and have an atheroprotective effect [41, 42]. A recent study showed that administration of ICA attenuates the secretion of tumour necrosis factor‐α, IL‐6 and IL‐1β in lipopolysaccharide‐treated RAW264.7 cells [13]. ICA also inhibits IL‐6 expression in chondrocytes challenged with IL‐1β [12]. Similar to these reports, we observed that treatment of THP‐1 macrophage‐derived foam cells with ICA decreased the expression and secretion of IL‐6 but increased the expression and secretion of IL‐10. At the same time, ICA downregulated the expression of M1 macrophage markers CD86 and iNOS but upregulated the expression of M2 macrophage markers CD206 and Arg‐1, suggesting that ICA can promote the phenotypic transition of macrophages from M1 to M2. In addition to lipid metabolism, HDAC9 contributes to inflammation [43, 44]. In this study, our results demonstrated that HDAC9 overexpression or miR‐1271‐5p silencing abolished the beneficial effects of ICA on the expression and secretion of IL‐6 and IL‐10, indicating the involvement of the miR‐1271‐5p/HDAC9 axis in ICA‐suppressed inflammatory response.

Although ICA has been shown to play a protective effect on macrophage lipid accumulation and inflammatory response through the miR‐1271‐5p/HDAC9 axis, there are several limitations present in our studies. Firstly, we do not observe the impact of ICA on these biological processes in animal models. Secondly, given the complexity of mechanisms of action of ICA, ICA may affect other pathophysiological processes involving atherogenesis. Thirdly, it is unclear whether ICA directly or indirectly modulates the expression of miR‐1271‐5p. Thus, further research is required to address these limitations and confirm its potential clinical applications.

In summary, we identify that ICA, as a critical microbially produced tryptophan metabolite, exerts a favourable effect on macrophage lipid accumulation and inflammatory response. Mechanistically, ICA activates the miR‐1271‐5p/HDAC9 signalling cascade to stimulate cholesterol efflux mediated by ABCA1 and ABCG1 as well as promotes M2 macrophage polarisation to antagonise inflammation. These findings offer novel mechanistical insights into the antiatherogenic action of ICA. Modifying intestinal microbiota to promote ICA biosynthesis or exogenous ICA administration could be new and promising strategies to prevent and treat atherosclerosis.

## Author Contributions

**Wei Luo:** writing – original draft (lead). **Jun Meng:** methodology (equal). **Xiao‐Hua Yu:** funding acquisition (equal), methodology (equal). **Zi‐Zhen Zhang:** data curation (lead), funding acquisition (equal). **Gang Wang:** formal analysis (lead), investigation (lead). **Jin He:** funding acquisition (equal), writing – review and editing (lead).

## Conflicts of Interest

The authors declare no conflicts of interest.

## Supporting information


Table S1

## Data Availability

The data that support the findings of this study are available from the corresponding author upon reasonable request.
