# Supplementary material for: Indole‐3‐Carboxaldehyde Inhibits Inflammatory Response and Lipid Accumulation in Macrophages Through the miR‐1271‐5p/HDAC9 Pathway
Source: J Cell Mol Med. 2024 Dec 19;28(24):e70263. doi: 10.1111/jcmm.70263 (PMC11656405; doi:10.1111/jcmm.70263)
Supplement: Supplementary file 1 — Table S1 [file JCMM-28-e70263-s001.docx]

Table S1 Sequences of primers for qRT-PCR

| Genes | Species | Primer sequences (5′ to 3′) |
| --- | --- | --- |
| CD36 | Human | AAGCCAGGTATTGCAGTTCTTT (Forward) |
|  |  | GCATTTGCTGATGT CTAGCACA (Reverse) |
| SR-A | Human | GCAGTG GGATCACTTTCACAA (Forward) |
|  |  | AGCTGTCATTGAGCGAGCATC (Reverse) |
| HMGCR | Human | GTCATTCCAGCCAAGGTTGT (Forward) |
|  |  | GGGACCACTTGCTTCCATTA (Reverse) |
| SREBP2 | Human | AGGAGAACATGGTGCTGA (Forward) |
|  |  | TAAAGGAGAGGCACAGGA (Reverse) |
| ABCA1 | Human | ACCCACCCTATGAACAACATGA (Forward) |
|  |  | GAGTCGGGTAACGGAAACAGG (Reverse) |
| ABCG1 | Human | ATTCAGGG ACCTTTCCTATTCGG (Forward) |
|  |  | CTCACCACTATTGAACTTCCCG (Reverse) |
| HDAC9 | Human | AGTAGAGAGGCATCGCAGAGA (Forward) |
|  |  | GGAGTGTCTTTCGTTGCTGAT (Reverse) |
| IL-6 | Human | ACTCACCTCTTCAGAACGAATTG (Forward) |
|  |  | CCATCTTTGGAAGGTTCAGGTTG (Reverse) |
| IL-10 | Human | TCTCCGAGATGCCTTCAGCAGA (Forward) |
|  |  | TCAGACAAGGCTTGGCAACCCA (Reverse) |
| CD86 | Human | CTGCTCATCTATACACGGTTACC (Forward) |
|  |  | GGAAACGTCGTACAGTTCTGTG (Reverse) |
| iNOS | Human | TCCAAGGTATCCTGGAGCGA (Forward) |
|  |  | CAGGGACGGGAACTCCTCTA (Reverse) |
| CD206 | Human | GGGTTGCTATCACTCTCTATGC (Forward) |
|  |  | TTTCTTGTCTGTTGCCGTAGTT (Reverse) |
| Arg-1 | Human | GTGGAAACTTGCATGGACAAC (Forward) |
|  |  | AATCCTGGCACATCGGGAATC (Reverse) |
| GAPDH | Human | TGTGGGCATCAATGGATTTGG (Forward) |
|  |  | ACACCATGTATTCCGGGTCAAT (Reverse) |
| miR-182-5p | Human | ATCACTTTTGGCAATGGTAGAACT (Forward) |
|  |  | TATGGTTTTGACGACTGTGTGAT (Reverse) |
| miR-383-5p | Human | AAGGTGATTGTGGCTGTCGT (Forward) |
|  |  | GTATCCAGTGCGTGTCGTGG (Reverse) |
| miR-27a-3p | Human | GCGCGTTCACAGTGGCTAAG (Forward) |
|  |  | AGTGCAGGGTCCGAGGTATT (Reverse) |
| miR-1271-5p | Human | CTTGGCACCTAGCAAGCACTCA (Forward) |
|  |  | GCGAGCACAGAATTAATACGAC (Reverse) |
| U6 | Human | CTCGCTTCGGCAGCACA (Forward) |
|  |  | AACGCTTCACGAATTTGCGT (Reverse) |
